# Supplementary material for: Superior Live Birth Rates, Reducing Sperm DNA Fragmentation (SDF), and Lowering Miscarriage Rates by Using Testicular Sperm Versus Ejaculates in Intracytoplasmic Sperm Injection (ICSI) Cycles from Couples with High SDF: A Systematic Review and Meta-Analysis
Source: Biology (Basel). 2025 Jan 26;14(2):130. doi: 10.3390/biology14020130 (PMC11851878; doi:10.3390/biology14020130)
Supplement: Supplementary file 1 [file biology-14-00130-s001.zip › Supplemental Table S1.pdf]

Supplementary Table S1. Summary of the outcome measures for each of the variables examined and their respective units.

Outcomes	Units	Estimator	CI (95%)	Nº. studies
DNA fragmentation	% of SDF	MD	-25.42 [-31.47; -17.30]	8
Fertilization	% (two pronuclei (2PN) zygotes	OR	OR=0.83 [0.67-1.03]	8
Clinical pregnancy	% (clinical pregnancy / ET)	OR	2.13 [1.35-3.36]	7
Miscarriage	% (pregnancy loss / clinical pregnancy)	OR	0.31 [0.14-0.70]	8
Live birth rate	% (live birth / ET)	OR	2.40 [1.32-4.36]	5

CI: confidence interval; DNA: Deoxyribonucleic acid; SDF: sperm DNA fragmentation; MD: mean difference; OR: odds ratio; ET: embryo transfer.
