# Supplementary material for: Superior Live Birth Rates, Reducing Sperm DNA Fragmentation (SDF), and Lowering Miscarriage Rates by Using Testicular Sperm Versus Ejaculates in Intracytoplasmic Sperm Injection (ICSI) Cycles from Couples with High SDF: A Systematic Review and Meta-Analysis
Source: Biology (Basel). 2025 Jan 26;14(2):130. doi: 10.3390/biology14020130 (PMC11851878; doi:10.3390/biology14020130)
Supplement: Supplementary file 1 [file biology-14-00130-s001.zip › Supplemental Table S2.pdf]

Supplementary Table S2. Quality assessment of all included studies.

Study	Criteria for nonrandomized estudies							
	Pre-intervention		Intervention	Post-intervention				Overall bias
	Bias due to confounding	Bias in selection of participants	Bias in classification of intervention	Bias due to deviations from intended interventions	Bias due to missing data	Bias in measurementof outcomes	Bias in selection of reported results	Risk of bias judgement
**Greco et al. 2005**	Moderate	Serious	Low	Low	Low	Moderate	Low	Serious
**Moskovtsev et al. 2010**	Moderate	Moderate	Low	Low	Low	Moderate	Low	Moderate
**Moskovtsev et al. 2012**	Moderate	Moderate	Low	Low	Low	Moderate	Low	Moderate
**Mehta et al. 2015**	Moderate	Serious	Low	Low	Low	Moderate	Low	Serious
**Esteves et al. 2015**	Moderate	Low	Low	Low	Low	Moderate	Low	Moderate
**Pabuccu et al. 2016**	Moderate	Moderate	Low	Low	Low	Low	Low	Moderate
**Bradley et al. 2016**	Moderate	Moderate	Moderate	Low	Low	Moderate	Low	Moderate
**Arafa et al. 2017**	Moderate	Moderate	Low	Low	Low	Low	Low	Moderate
**Herrero et al. 2019**	Moderate	Low	Low	Low	Low	Low	Low	Low
**Alharbi et al. 2020**	Moderate	Low	Moderate	Low	Low	Low	Low	Low
**Xie et al. 2020**	Moderate	Serious	Moderate	Low	Moderate	Moderate	Moderate	Serious
**Zhou et al. 2024**	Moderate	Moderate	Moderate	Low	Low	Moderate	Low	Moderate
