# Supplementary figures and images for: Superior Live Birth Rates, Reducing Sperm DNA Fragmentation (SDF), and Lowering Miscarriage Rates by Using Testicular Sperm Versus Ejaculates in Intracytoplasmic Sperm Injection (ICSI) Cycles from Couples with High SDF: A Systematic Review and Meta-Analysis
Source: Biology (Basel). 2025 Jan 26;14(2):130. doi: 10.3390/biology14020130 (PMC11851878; doi:10.3390/biology14020130)

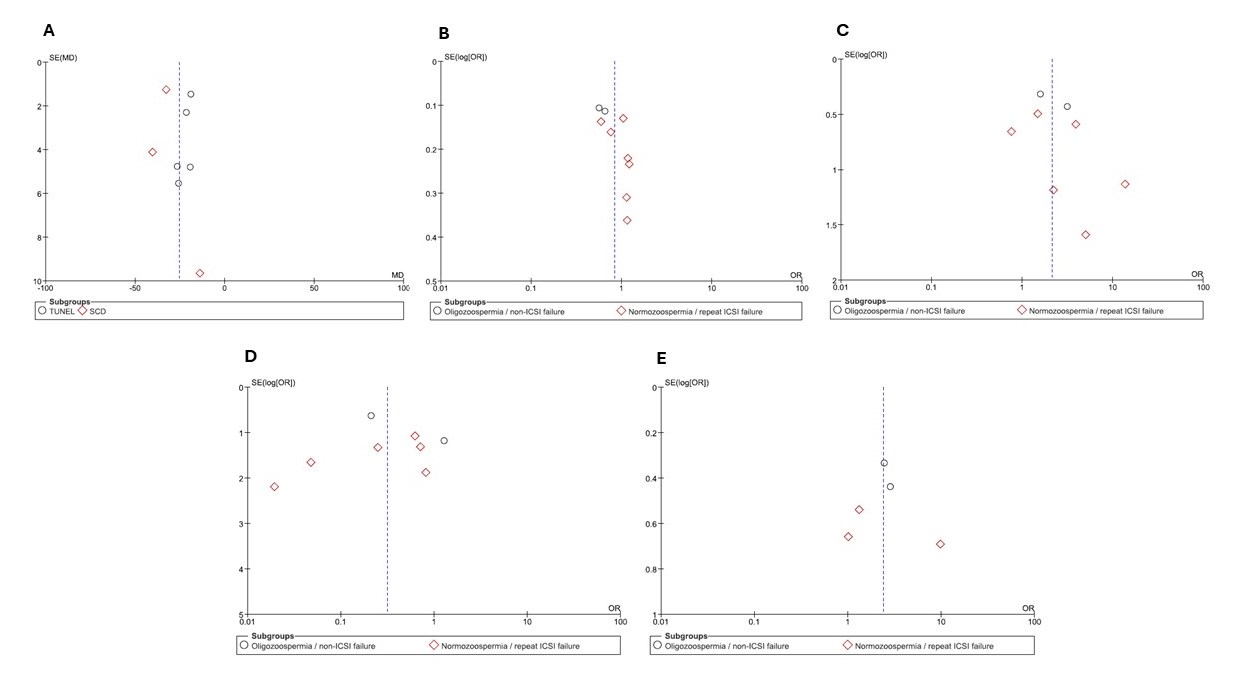

Supplement: Supplementary file 1 [file biology-14-00130-s001.zip › Supplemental Figure S1.jpg]
